# The impact of COVID-19 on cryptocurrency markets: A network analysis based on mutual information

**DOI:** 10.1371/journal.pone.0259869

**Published:** 2022-02-18

**Authors:** Mi Yeon Hong, Ji Won Yoon

**Affiliations:** School of Cybersecurity & Institute of Cybersecurity and Privacy (ICSP), Korea University, Seoul, Republic of Korea; URV: Universitat Rovira i Virgili, SPAIN

## Abstract

The purpose of our study is to figure out the transitions of the cryptocurrency market due to the outbreak of COVID-19 through network analysis, and we studied the complexity of the market from different perspectives. To construct a cryptocurrency network, we first apply a mutual information method to the daily log return values of 102 digital currencies from January 1, 2019, to December 31, 2020, and also apply a correlation coefficient method for comparison. Based on these two methods, we construct networks by applying the minimum spanning tree and the planar maximally filtered graph. Furthermore, we study the statistical and topological properties of these networks. Numerical results demonstrate that the degree distribution follows the power-law and the graphs after the COVID-19 outbreak have noticeable differences in network measurements compared to before. Moreover, the results of graphs constructed by each method are different in topological and statistical properties and the network’s behavior. In particular, during the post-COVID-19 period, it can be seen that Ethereum and Qtum are the most influential cryptocurrencies in both methods. Our results provide insight and expectations for investors in terms of sharing information about cryptocurrencies amid the uncertainty posed by the COVID-19 pandemic.

## Introduction

Recent advances in science and technology have created large data sets in a variety of fields, and we live in a complex world where they are interconnected. Thus, complex network analysis has become a powerful method, and it provides a useful map that describes a wide range of systems of high technological and intellectual importance [1]. Fundamentally, a complex network is composed of two basic components: nodes that are elements of the system and edges that represent the pairwise relationships between those elements. Therefore, these components describe complicated real-world systems from different and complementary perspectives and these are a new and rich source of domain-specific information [2]. Various studies about complex networks have been performed in social interactions [3], communication networks [4], biological networks [5], transportation infrastructures [6], and so on. In particular, after Mantegna’s [7] groundbreaking research, complex network theory has been successfully implemented in the financial market, and various empirical analyzes of the stock market have been conducted based on this theory [8–11].

Cryptocurrency network is a research field that has grown rapidly as recently observed data has been secured. Each cryptocurrency is a node of this type of network, but most are still unexplored. Nakamoto [12] first proposed a new form of asset, Bitcoin, in 2008. After that, active trading began in 2013, and various cryptocurrencies began to appear, therefore, the cryptocurrency market is rather young. Compared to existing stock market analysis, there is little data analysis on cryptocurrencies traded in the cryptocurrency market. In essence, stock price and cryptocurrency price data have random characteristics in time-series, but they are differentiated in the following points. Cheung et al. [13], Carrick [14], and Liu and Serletis [15] show that the cryptocurrency market is much more volatile than the stock market. Also, the price of other cryptocurrencies is affected by the price of a specific cryptocurrency [16, 17]. Some studies consider the relationship between cryptocurrencies and a variety of other financial assets, mostly for Bitcoin [18–20]. Similarly, the few studies examining the relationship among cryptocurrencies are those by Osterrieder et al. [21] and Bouri et al. [22]. Particularly, Gandal and Halaburda [23] analyzed the relationship between various cryptocurrencies through winner-take-all dynamics. Furthermore, anomalies in the cryptocurrency market are investigated by Kurihara and Fukushima [24], and Caporale and Plastun [25].

The complexity of the cryptocurrency market can be studied from various perspectives and the purpose of our study is to construct a network based on cryptocurrency market data to find potential relationships and effects between more types of cryptocurrencies. Cryptocurrency network analysis can provide a deeper and more complex picture of the cryptocurrency market as a whole as it helps to understand the interaction between the variables. There was a special event called COVID-19 outbreak between 2019 and 2020 when our study was conducted. Even though various cryptocurrency analysis studies have been executed based on this event, most of the literature related to cryptocurrency focuses on Bitcoin or small cryptocurrency groups [26–29] and analyzes various cryptocurrency properties about other markets without taking into account the internal dynamic evolution of the cryptocurrency market as a whole [30–32]. Our main consideration through this study is an attempt to investigate how COVID-19 affects the structure and dynamics of the cryptocurrency market. To this end, we aim to analyze the structure of the market and the collective behavior of its entities based on the Minimum Spanning Tree (MST) [33–36] and Planar Maximally Filtered Graph (PMFG) [37] to which Pearson’s correlation coefficient (hereafter referred to as correlation coefficient) and mutual information are applied.

Studies of associations with given datasets in various fields have generally been based on the correlation coefficient [38] that is a long-standing measure of the strength of statistical dependence. It is useful to determine the relationship between two random variables because the correlation coefficient shows how currencies work together in new and volatile markets, but this approach can only measure linear relationships [39]. We note that to include nonlinear dependencies, one could alternate Spearman’s rank correlation instead of the correlation coefficient. However, the problem with Spearman’s rank correlation approach is that it considers only a limited kind of association pattern, such as monotonically increasing function [40]. On the other hand, mutual information [41] does not have such restrictions. Mutual information is a measure of statistical dependence between two random variables that are sampled simultaneously, and it is a more general approach for measuring non-linear relationships. It can be used to identify the relationship between datasets that are not detected by the commonly used linear measure of correlation. Academic research provides extensive evidence of non-linearities in financial and cryptocurrency markets [42–46]. This approach provides an analysis of the possibilities of cryptocurrency portfolios to a broader scope. Also, another distinctive difference between the correlation coefficient and mutual information is that the latter can be applied in numerical sequences and symbolic sequences, but the former can only be used on numerical sequences. So, the correlation coefficient only renders a single number for the entire time period, it does not account for changes over time. Mutual information that utilizes symbol sequences is thus a natural alternative to the correlation coefficient function.

The key contributions of this study are listed as follows:

- First, to grasp cryptocurrency log-returns characteristics, we use Symbolic Time Series Analysis (STSA) to divide the original time series data into a finite number of intervals. The newly generated discrete values become a tool for calculating mutual information.
- Then we discuss the computation of mutual information and correlation coefficient for pre- and post-COVID-19 outbreaks respectively. We create a distance matrix based on each method, which identifies statistical characteristics.
- In consideration of the above-mentioned limitations, we aim to construct cryptocurrency networks using MST and PMFG and analyze their topological dynamics, and behavior of the network to compare how the COVID-19 outbreak has changed the relationship among cryptocurrencies.

**Fig 1:**
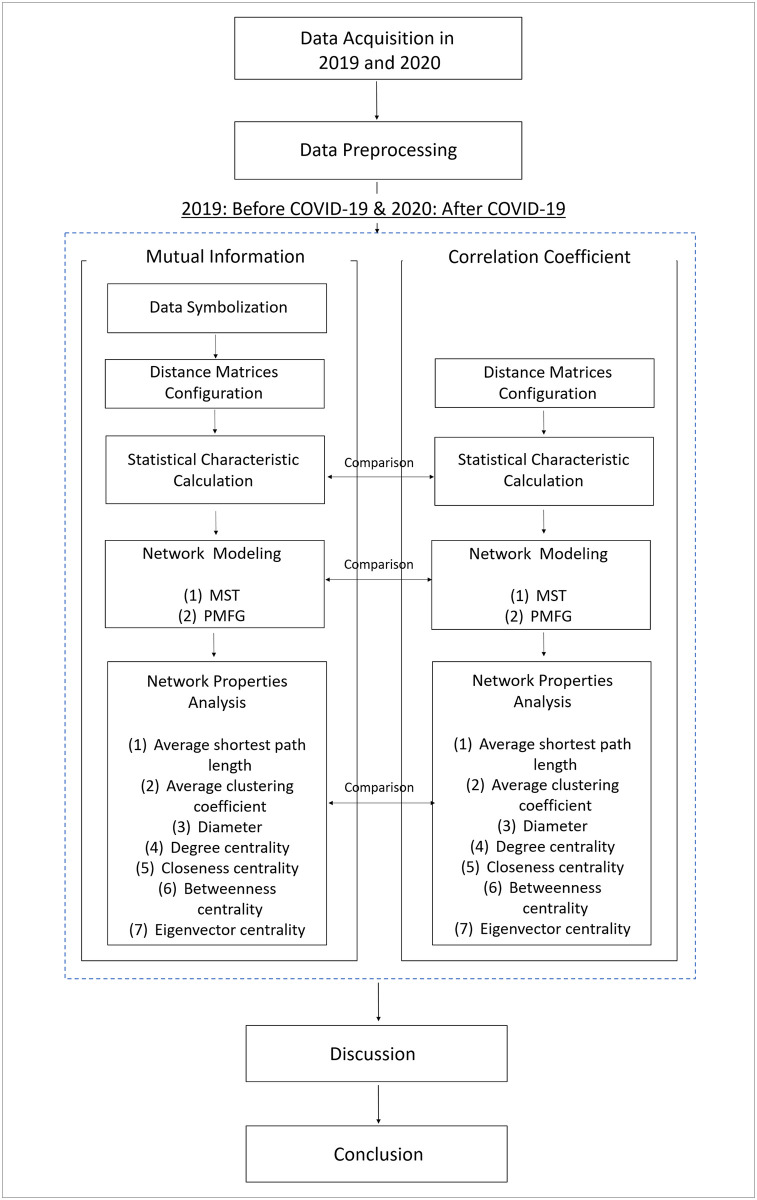
Research overview.

Through a comprehensive analysis of topological dynamics and market properties, we revealed that various changes have occurred in the cryptocurrency market due to the influence of the COVID-19 outbreak. We also concluded that the approach through mutual information is more effective in identifying these changes. To the best of our knowledge, this is the first effort in the literature for network analysis, using linear and non-linear approaches to investigate the effect of COVID-19 on the cryptocurrency market behavior during 2019 and 2020. The overview of the methodology conducted for this study is described in Fig 1.

## Background

### Mutual information

Mutual information is close ties to Shannon’s entropy [47]. Consider a random variable *X* with probability distribution *p*(*x*) ≡ *p*(*X* = *x*). In the discrete case, the Shannon’s entropy is defined as

$$\begin{array}{c} H\left(X\right)=-\sum_{x}p\left(x\right)\text{log}\;p\left(x\right). \end{array}$$ (1)

Now we consider two random variables, *X* and *Y*, and let *p*(*x*, *y*) denote their joint probability distribution. The joint entropy for *X* and *Y* is given by

$$\begin{array}{c} H\left(X,Y\right)=-\sum_{x,y}p\left(x,y\right)\text{log}\;p\left(x,y\right). \end{array}$$ (2)

We can also define conditional entropy of *Y* given *X* (or vice versa), as follows

$$\begin{array}{c} H\left(Y|X\right)=-\sum_{x}p\left(x\right)[\sum_{y}p\left(y|x\right)\text{log}\;p\left(y|x\right)]. \end{array}$$ (3)

Then the mutual information of discrete random variables *X* and *Y* is

$$\begin{array}{c} I\left(X;Y\right)=\sum_{x,y}p\left(x,y\right)log\frac{p(x,y)}{p(x)p(y)}. \end{array}$$ (4)

It is directly related to Shannon’s entropy, thus, it can be expressed equivalently using entropies

$$\begin{array}{cc} I(X;Y) & =H(X)+H(Y)-H(X,Y)\\ & =H(Y)-H(Y|X)\\ & =H(X)-H(X|Y). \end{array}$$ (5)

Mutual information is always greater than or equal to zero, with equality iff *X* and *Y* are independent.

### Hierarchical networks

Then we construct two types of filtered graphs to show the hierarchical arrangement of a portfolio of cryptocurrencies. MST is the primary method used in our research. Let *G*(*V*, *E*) be an undirected graph which is connected and acyclic, where *V* = {*v*_1_, …, *v*_*n*_} is the set of nodes, *E* = {*e*_1_, …, *e*_*m*_} is the set of edges. It connects all nodes of *G*(*V*, *E*), so if the number of nodes is *V* = *n* then the number of edges is *E* = *n* − 1. For a graph *G*(*V*, *E*) with positively weighted edges, we adopt MST the spanning graph with a minimum total cost of weights. The distance between the two currencies mentioned above is used to determine the weight of MST. In our paper, Kruskal’s algorithm is applied as a method. The Kruskal algorithm is a greedy algorithm that ranks the weights of edges in ascending order and adds the next edge with the smallest weight to the current set of MST if this addition does not create a cycle. Finally, the complete graph on *V* nodes has $\frac{n(n-1)}{2}$ edges and the complexity of the Kruskal algorithm is *O*(*E* log(*V*)), which guarantees a connected tree without cycles and planar.

The MST filters a significant amount of valid information of a corresponding network because it only has the edges needed to connect the shortest distance. The PMFG method is an extension of the MST, used as a complementary method to overcome the non-unique deficiency of MST and dig up the fundamental information about a cryptocurrency network. PMFG is a network drawn in a plane such that there are no intersecting links, and it has 3(*n* − 2) edges. Its complexity is *O*(*V*^3^). Therefore, the PMFG preserves more useful information in some parts that might be missed in MST. With both PMFG and MST, we can better understand the nature of the network.

### Network tolopogical properties

Additionally, we can interpret the network’s behavior by applying some useful measurements to analyze the configured MST and PMFG. One basic but essential measure in network analysis is well-established centrality. Centrality is an indicator of the degree to which a particular node is centered in the entire network, and the node located at the center exerts a strong influence on decision-making within the network. Thus, the centrality of a node is a measure of the potential importance, influence, prominence of the node in a network that is derived from its relative position compared to other nodes in a network. These measurements analyze the topological characteristics of the networks and provide us with a better understanding of MSTs. There are primarily four methods employed to figure out the roles of specific nodes in the networks, e.g. degree centrality, closeness centrality, betweenness centrality, and eigenvector centrality. Apart from these, it is also possible to obtain information for network analysis by using the average shortest path length, average cluster coefficient, and diameter.

- Average shortest path length: It is the mean of the steps along the shortest paths for all pairs of nodes,
  $$\begin{array}{c} L=\sum_{s,t\in V}\frac{d(s,t)}{n(n-1)} \end{array}$$ (6)
  where *V* is the set of nodes in the graph and *d*(*s*, *t*) is the shortest path from *s* to *t*.
- Average clustering coefficient: It is the mean measurement of the degree to which nodes in a graph tend to cluster together,
  $$\begin{array}{c} C=\frac{1}{n}\sum_{v\in G}c_{v},c_{v}=\frac{2T(v)}{deg(v)(deg(v)-1)} \end{array}$$ (7)
  where *T*(*v*) is the number of triangles through node *v*, *deg*(*v*) is the degree of *v*, and *n* is the number of nodes in *G*.
- Diameter: It is the maximum of all node eccentricities in a network,
  $$\begin{array}{c} D=\underset{st}{\text{max}}\;d(s,t). \end{array}$$ (8)
- Degree centrality: It identifies the importance of a node by the number of links incident upon a node, and if one node has many connections with the other, it is located in the center of the network. To calculate the degree centrality *C*_*d*_(*x*) of a node *x* for a given network, the following formula is used,
  $$\begin{array}{c} C_{d}\left(x\right)=\frac{c_{d}\left(x\right)}{n-1}. \end{array}$$ (9)
  The *c*_*d*_(*x*) is node degree and *n* is the number of nodes used in the formula above.
- Closeness centrality: It is defined as the average length of the shortest path between the node and all other nodes in the graph. It reflects the node’s ability to access information and how fast a node can connect to other nodes in the network, and can be expressed as
  $$\begin{array}{c} C_{c}\left(u\right)=\frac{n-1}{\sum_{v}^{n-1}d\left(v,u\right)} \end{array}$$ (10)
  where *d*(*v*, *u*) expresses the shortest path distance between nodes *v* and *u*.
- Betweenness centrality: It is the degree of a node being among other nodes in the network and indicates to what extent a node is in direct connection with nodes that are not directly linked to each other. In other words, it represents the degree to which a node performs a role of a bridge
  $$\begin{array}{c} C_{b}\left(v\right)=\sum_{s,t\in V}\frac{\sigma (s,t|v)}{\sigma (s,t)} \end{array}$$ (11)
  where *V* is the total number of nodes, *σ*(*s*, *t*) is the sum of the shortest path between *s*, *t*, and *σ*(*s*, *t*|*V*) is the sum of those paths passing through some node *v* other than *s*, *t*.
- Eigenvector centrality: It is an extension of the concept of degree centrality. It assigns voting scores to all nodes in the network based on the idea that connections to high-scoring nodes contribute more to the score of the node than connections to low-scoring nodes. Adjacency matrix is used to obtain the centrality score, let *A* = (*a*_*v*,*t*_) be the adjacency matrix and *a*_*v*,*t*_ = 1 if node *v* is linked to node *t*, otherwise *a*_*v*,*t*_ = 0. The relative centrality *x*, the score of node *v* can be represented as
  $$\begin{array}{c} x_{v}=\frac{1}{\lambda }\sum_{t\in M_{(}v)}x_{t}=\frac{1}{\lambda }\sum_{t\in G}a_{v,t}x_{t} \end{array}$$ (12)
  where *M*_(_ *v*) is a set of the neighbors of *v* and λ is a constant called eigenvalue. With a slight rearrangement, this is an eigenvector equation, which can be rewritten in vector notation, *Ax* = λ*x*.

## Network construction

### Data description

There are thousands of cryptocurrencies traded on hundreds of platforms around the world. We use *CoinMarketCap* [48], one of the cryptocurrency industry utilities that keeps the records of all cryptocurrencies traded on numerous cryptocurrency exchanges, aggregates, and reports recently traded prices. For each currency, it reports the total value of the outstanding currency, total trading volume, and rank by trading volume over the past month and the past 24 hours [49]. As the cryptocurrency market grows every year, new cryptocurrencies are being added. Therefore, there were many cryptocurrencies that were insufficient to collect data.

Only cryptocurrencies with a sufficiently long time are selected for analysis, as they can last anywhere from months to years. The experimental period was set from Jan 1, 2019, to Dec 31, 2020, and the close price data of 731 days were obtained for 102 cryptocurrencies. Table 1 shows the list used in our research. The types of cryptocurrencies are divided into 29 coins that are minable, 27 coins that are non-minable, 44 Ethereum tokens, and 2 Binance chain tokens according to each technical difference.

**Table 1:** Cryptocurrency list.

Symbol	Name	Type	Symbol	Name	Type
ZRX	0x	Ethereum token	LSK	Lisk	Non-mineable coin
ABBC	ABBC Coin	Mineable coin	LTC	Litecoin	Mineable coin
ELF	aelf	Ethereum token	LRC	Loopring	Ethereum token
ANT	Aragon	Ethereum token	MAID	MaidSafeCoin	Ethereum token
ARDR	Ardor	Non-mineable coin	MKR	Maker	Ethereum token
ARK	Ark	Non-mineable coin	MONA	MonaCoin	Mineable coin
REP	Augur	Ethereum token	XMR	Monero	Mineable coin
BNT	Bancor	Ethereum token	NANO	Nano	Non-mineable coin
BAT	Basic Attention Token	Ethereum token	XEM	NEM	Non-mineable coin
BNB	Binance Coin	Mineable coin	NEO	Neo	Non-mineable coin
BTC	Bitcoin	Mineable coin	NEXO	Nexo	Binance chain token
BCH	Bitcoin Cash	Mineable coin	NMR	Numeraire	Ethereum token
BCD	Bitcoin Diamond	Mineable coin	OMG	OMG Network	Ethereum token
BTG	Bitcoin Gold	Mineable coin	ONT	Ontology	Non-mineable coin
BTS	BitShares	Non-mineable coin	TRAC	Origin Trail	Ethereum token
BTM	Bytom	Mineable coin	PAX	Paxos Standard	Ethereum token
ADA	Cardano	Mineable coin	PNT	Penta	Ethereum token
CEL	Celsius	Ethereum token	NPXS	Pundi X	Ethereum token
LINK	Chainlink	Ethereum token	QTUM	Qtum	Non-mineable coin
CVC	Civic	Ethereum token	QNT	Quant	Ethereum token
CRO	Crypto.com Coin	Ethereum token	QKC	QuarkChain	Ethereum token
CVT	Cyber Vein	Ethereum token	RVN	Ravencoin	Mineable coin
DASH	Dash	Mineable coin	REN	Ren	Ethereum token
MANA	Decentraland	Ethereum token	REV	Revain	Ethereum token
DCR	Decred	Mineable coin	SC	Siacoin	Non-mineable coin
DGB	DigiByte	Mineable coin	SNT	Status	Ethereum token
DIVI	Divi	Mineable coin	STEEM	Steem	Non-mineable coin
DOGE	Dogecoin	Mineable coin	STORJ	Storj	Ethereum token
DX	DxChain Token	Ethereum token	STRAX	Stratis	Non-mineable coin
ETN	Electroneum	Mineable coin	CHSB	SwissBorg	Ethereum token
NRG	Energi	Mineable coin	SNX	Synthetix Network Token	Ethereum token
ENJ	Enjin Coin	Ethereum token	USDT	Tether	Ethereum token
EOS	EOS	Non-mineable coin	XTZ	Tezos	Non-mineable coin
ETH	Ethereum	Mineable coin	TMTG	The Midas Touch Gold	Ethereum token
ETC	Ethereum Classic	Mineable coin	THETA	THETA	Non-mineable coin
FTM	Fantom	Non-mineable coin	TOMO	TomoChain	Mineable coin
FIL	Filecoin	Mineable coin	TRX	TRON	Non-mineable coin
GNO	Gnosis	Ethereum token	TUSD	TrueUSD	Binance chain token
GLM	Golem	Ethereum token	UBT	Unibright	Ethereum token
XHV	Haven Protocol	Non-mineable coin	USDC	USD Coin	Ethereum token
HOT	Holo	Ethereum token	UTK	Utrust	Ethereum token
ZEN	Horizen	Mineable coin	VET	VeChain	Non-mineable coin
HT	Huobi Token	Ethereum token	XVG	Verge	Mineable coin
ICX	ICON	Non-mineable coin	WAN	Wanchain	Non-mineable coin
RLC	iExec RLC	Ethereum token	WAVES	Waves	Non-mineable coin
IOST	IOST	Non-mineable coin	WAXP	WAX	Non-mineable coin
MIOTA	IOTA	Non-mineable coin	XRP	XRP	Non-mineable coin
KIN	Kin	Non-mineable coin	ZB	ZB Token	Ethereum token
PNK	Kleros	Ethereum token	ZEC	Zcash	Mineable coin
KMD	Komodo	Mineable coin	ZIL	Zilliqa	Mineable coin
KCS	KuCoin Shares	Ethereum token	KNC	Kyber Network	Ethereum token

### Data preprocessing

In order to evaluate the correlation and mutual information for each pair of nodes, we first transform the time series of closing prices of 102 cryptocurrencies to the log ratios between consecutive daily closing prices. We use logarithmic-return, which is by far the most widely used in the stock market. Considering a network of *C* cryptocurrencies with closing prices in *T* training days, we denote $P_{i}^{t}$ and $R_{i}^{t}$ as the closing price and logarithmic return of cryptocurrency *i* at day *t*, and it is defined as

$$\begin{array}{c} R_{i}^{t}=lnP_{i}^{t}-lnP_{i}^{t-1} \end{array}$$ (13)

where *i* = {1, …, *C*} and *t* = {2, …, *T*}.

### Time series symbolization

**Fig 2:**
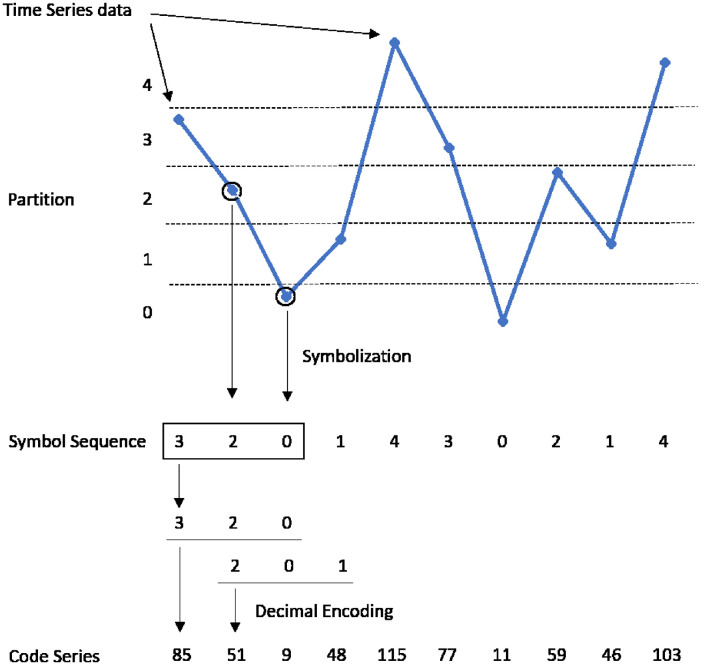
Process of time series symbolization.

In this paper, we propose to use a time series symbolization analysis method based on [50]. Recognizing temporal patterns in complex dynamic processes requires a language to express and analyze these patterns, and data encoding seems to be a very effective way to introduce such a language. Through a data encoding process in which the values of the given original time series data are converted into a finite set of symbols that yield a finite string, the actual signal is replaced with a symbolic representation. Fig 2 illustrates the overall process in an easy-to-understand manner and this can be achieved through the following steps.

- For a given time series data, symbolize the data corresponding to the interval. The number of possible symbols, *n*, is termed the symbol set size.
- Then words(or symbol sequences) are constructed from the *l* successive symbol values occurring at each point in time and the *l*-step template slides along with the symbol series.
- Each possible word is represented by a corresponding decimal value determined by the position of each symbol in the template to form a new series, called code series.

When a series of symbols are converted to indicate a characteristic, it is important to determine the length of the word. If *l* is too small, some significant information will diminish, reducing the practical importance of statistical results. Conversely, the frequency of each word is too small to obtain reliable statistical results, if *l* is too big. Thus, when dealing with our finite data, a trade-off of the length of *l* is required. To ensure that the probability of occurrence of each symbol is equal for all sizes of the symbol set *n*, we adopt *n* = 5. All returns consist of five symbols, that form an equiprobable binning scheme, each represented respectively by 0,1,2,3,4 as follows:

$$\begin{array}{c} S_{i}^{t}=\{\begin{array}{cc} 0 & \text{for}\;R_{i}^{t}\leq q_{0.2}\\ 1 & \text{for}\;q_{0.2}<R_{i}^{t}\leq q_{0.4}\\ 2 & \text{for}\;q_{0.4}<R_{i}^{t}\leq q_{0.6}\\ 3 & \text{for}\;q_{0.6}<R_{i}^{t}\leq q_{0.8}\\ 4 & \text{for}\;q_{0.8}<R_{i}^{t}\; \end{array} \end{array}$$ (14)

Through this process, each element of $R_{i}^{t}$ is transformed to the $S_{i}^{t}$, therefore, we create a new sequence of numbers belonging to it. Symbols indicate that return 0 is in the low, 2 is in the middle, and 4 is in the high ranges. After symbolization, we obtain *N* length of symbol series $S_{i}^{t}=S_{i}^{0},S_{i}^{1},\ldots ,S_{i}^{N-1}$. We take *l* = 3 for each return symbolic series. A method for converting words formed by 3-step sliding templates along the symbol series into decimal values is by the following formula

$$\begin{array}{c} M_{i}^{t}=S_{i}^{t+l-1}\times n^{0}+S_{i}^{t+l-2}\times n^{1}+\ldots +S_{i}^{t}\times n^{l-1} \end{array}$$ (15)

where *t* = {2, 3, …, *N* − *l*}. A combination of symbol set size and word length satisfies the total possible number of sequences *N* that can be represented,

$$\begin{array}{c} N=n^{l}. \end{array}$$ (16)

### Distance metrics using mutual information

We use two different approaches to build MSTs, to explore the results. As discussed in the introduction, most of the few studies on cryptocurrencies have only dealt with correlation coefficient analysis to establish the distance between nodes in the network. We adopt the mutual information to find non-linear dependencies in cryptocurrency markets using the symbolized data that we have created in the previous section.

Using the decimal value *M* of the cryptocurrencies *i* and *j* at day *t*, we can approximately compute the joint entropy by $H(M_{i}^{t},M_{j}^{t})$, and the mutual information is estimated by

$$\begin{array}{c} I\left(M_{i}^{t};M_{j}^{t}\right)=H\left(M_{i}^{t}\right)+H\left(M_{j}^{t}\right)-H\left(M_{i}^{t},M_{j}^{t}\right). \end{array}$$ (17)

When computing the mutual information using Shannon’s entropy, we can set the number of bins. Once the bin number is adequately large, an additional increase in the number of bins no longer affects the accuracy of mutual information. Thus, we choose 10 × 10 bins in this study.

To obtain a distance between nodes, we convert mutual information to a distance metric *D*_*I*_ which is called normalized distance function [51] by

$$\begin{array}{c} D_{I}\left(X,Y\right)=1-\frac{I(X,Y)}{H(X,Y)} \end{array}$$ (18)

*D*_*I*_ follows 0 ≤ *D*_*I*_(*X*, *Y*) ≤ 1 for all cryptocurrencies *X* and *Y*. For a function *D*_*I*_ to be a distance, it must satisfies (i) *D*_*I*_(*X*, *Y*) > 0 for *X* ≠ *Y*, (ii) *D*_*I*_(*X*, *X*) = 0, (iii) *D*_*I*_(*X*, *Y*) = *D*_*I*_(*Y*, *X*), (iv) *D*_*I*_(*X*, *Y*) ≤ *D*_*I*_(*X*, *Z*) + *D*_*I*_(*Z*, *Y*) for all pairs (*X*, *Y*). Thus, the distance of cryptocurrencies *i* and *j* in the network is

$$\begin{array}{c} D_{I}\left(M_{i}^{t},M_{j}^{t}\right)=1-\frac{I(M_{i}^{t},M_{j}^{t})}{H(M_{i}^{t},M_{j}^{t})}. \end{array}$$ (19)

### Distance metrics using correlation coefficient

We also compute correlation coefficient over the same period. It defines degree of similarity between the synchronous time evolution of a pair of cryptocurrency prices

$$\begin{array}{c} \rho_{i,j}=\frac{\sum_{t=1}^{T}\left(R_{i}^{t}-{\bar{R}}_{i}\right)\left(R_{j}^{t}-{\bar{R}}_{j}\right)}{\sqrt{\sum_{t=1}^{T}{\left(R_{i}^{t}-{\bar{R}}_{i}\right)}^{2}}\sqrt{\sum_{t=1}^{T}{\left(R_{j}^{t}-{\bar{R}}_{j}\right)}^{2}}} \end{array}$$ (20)

where ${\bar{R}}_{i}$ is the average log-return of cryptocurrency *i* over *T* trading days. For 102 cryptocurrencies, this results in a 102 × 102 matrix with all entries within the interval [−1, 1].

To transform the correlation matrix into a distance matrix, a metric can be defined using a distance function, denoted by

$$\begin{array}{c} d_{\rho }\left(X,Y\right)=\sqrt{2(1-\rho_{X,Y})} \end{array}$$ (21)

where *d*_*ρ*_(*X*, *Y*) is in the interval [0, 2], since *ρ*_*X*,*Y*_ range is [-1,1]. With this method, *d*_*ρ*_(*X*, *Y*) fulfills three axioms of an Euclidean metric in the following manner (i) *d*_*ρ*_(*X*, *Y*) = 0 if and only if *X* = *Y*, (ii) *d*_*ρ*_(*X*, *Y*) = *d*_*ρ*_(*Y*, *X*), and (iii) *d*_*ρ*_(*X*, *Y*) < *d*_*ρ*_(*X*, *Z*) + *d*_*ρ*_(*Z*, *Y*) for all practical purpose.

## Results

### Comparative study of the methods

**Fig 3:**
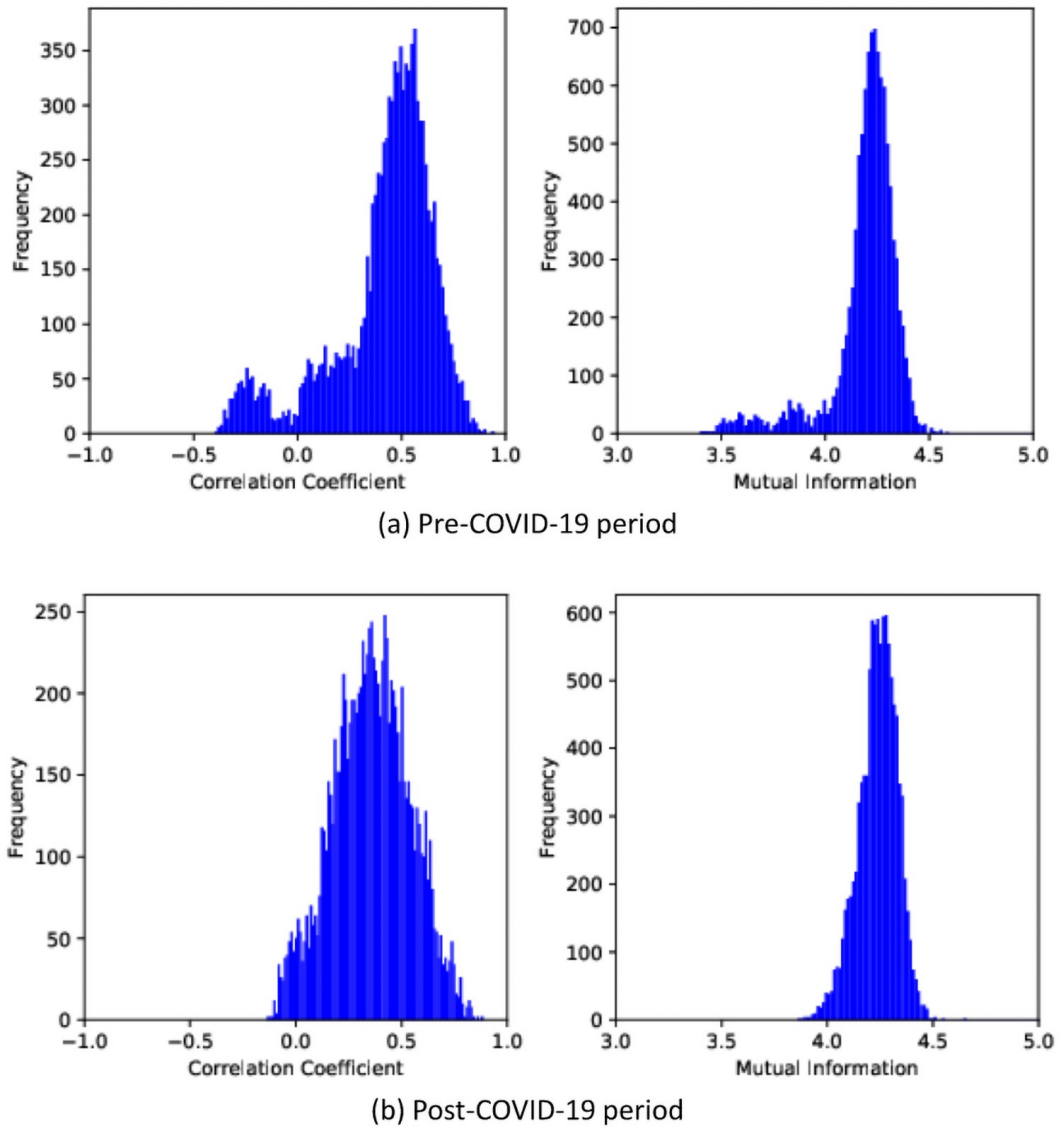
Histogram of correlation coefficient and mutual information in pre- and post-COVID-19 periods. (a) Frequency distribution of two methods in 2019. (b) Frequency distribution of two methods in 2020.

To analyze how effective the mutual information is compared to the correlation coefficient, we first observe the frequency distribution of corresponding mutual information values and correlation coefficient values of all pairs of cryptocurrencies in Fig 3. During the pre-COVID-19 period, all the correlation coefficient values fall into the interval [-0.14, 0.89] and the majority of the values exist between 0.12 and 0.64. Also, 396 pairs of cryptocurrencies have a strong positive linear relationship that is above 0.7, but 348 pairs of cryptocurrencies have a weak negative relationship. For the mutual information method, all the values fall into the interval [3.92, 4.49] and the majority of the values exist between 4.14 and 4.37. During the post-COVID-19 period, all the correlation coefficient values fall into the interval [-0.40, 0.95] and the majority of the values exist between 0.31 and 0.72. Also, 742 pairs of cryptocurrencies have a strong positive linear relationship that is above 0.7, but 822 pairs of cryptocurrencies have a negative relationship and most of them are weak. For the mutual information method, all the values fall into the interval [3.39, 4.60] and the majority of the values exist between 4.10 and 4.37.

**Fig 4:**
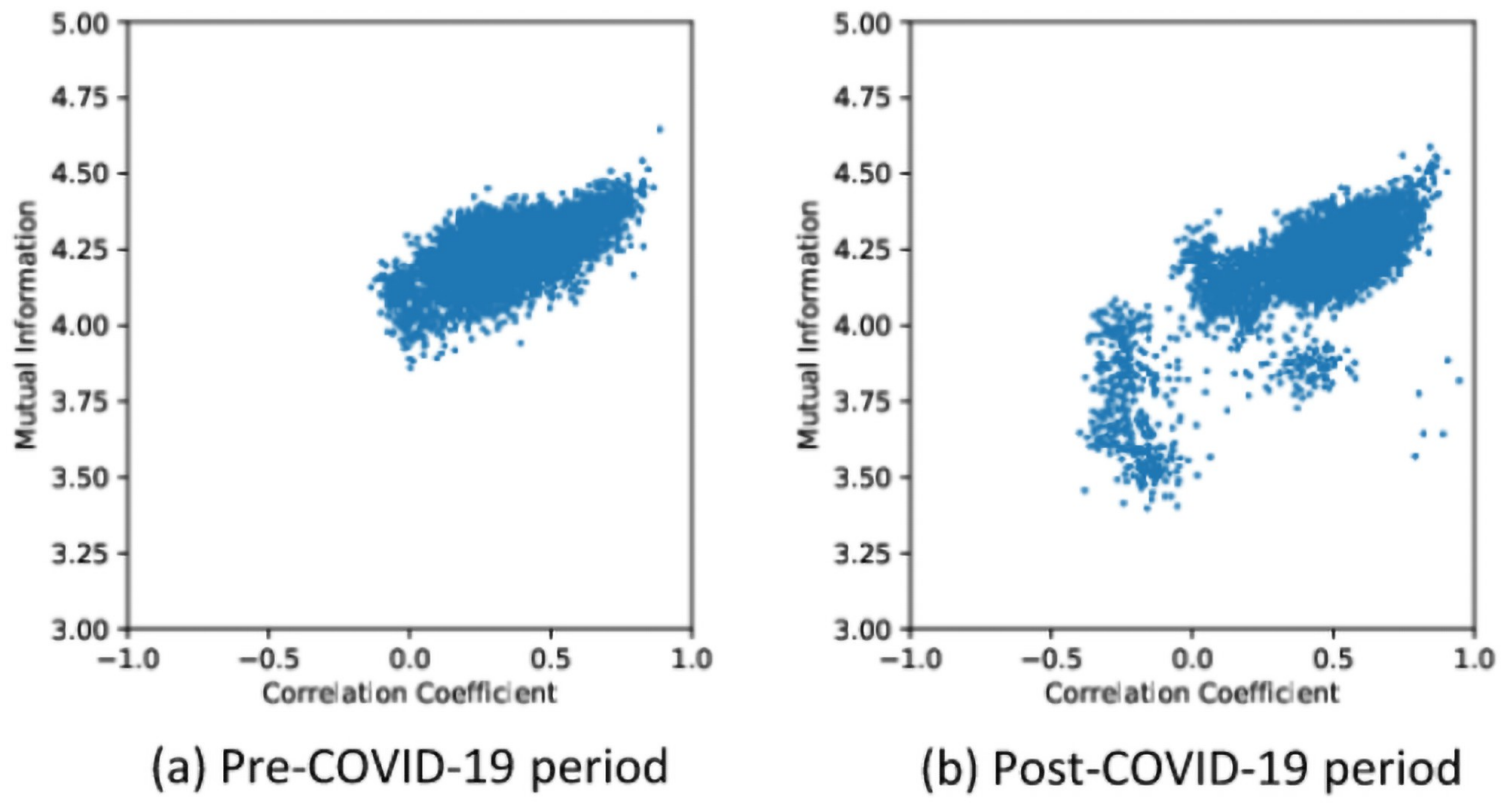
Comparison of correlation coefficient and mutual information over a given period.

After then, the overall linearity in Fig 4 suggests that the non-linear method based on mutual information not only managed to capture linear relationships but also capture non-linearity found in the data which the method based on correlation coefficient failed to capture. In both periods, it is observed that cryptocurrency pairs with high mutual information are much more pronounced at higher correlation coefficient levels compared to low or zero correlation coefficient values. In addition, during the period after COVID-19 outbreak, it can be seen that the scatter plot between the data spreads wider and deeper. This indicates that mutual information and correlation coefficient values have decreased in many cryptocurrencies since post-COVID-19, and from this result, there are substantial variations in the relationship between cryptocurrency pairs derived from the two measures.

### Statistics of correlation coefficient and mutual information based distance matrix

**Fig 5:**
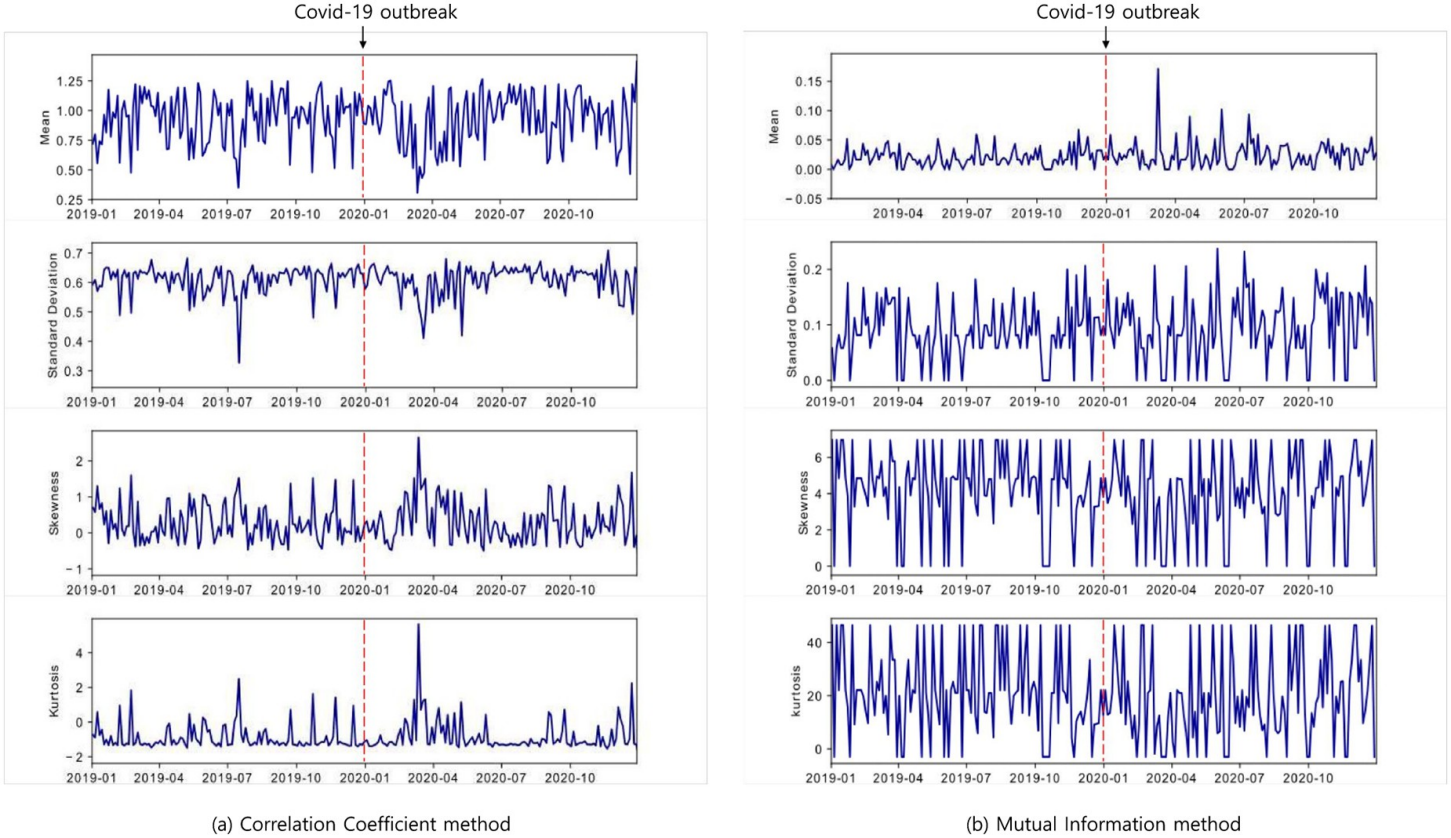
The mean, standard deviation, skewness, and kurtosis of distance matrix of correlation coefficient and mutual information methods at each time step.

Before constructing networks, we first examine the statistical properties of the correlation coefficient and mutual information-based distance matrix for 102 currencies. Fig 5 shows the dynamically evolving graphs for four descriptive statistics of both methods during pre- and post-COVID-19 periods, respectively. In mutual information theory, the 3-step template slides, which are used to symbolize the time-series data, are applied equally for correlation coefficient-based statistics measurements.

From each figure, it can be founded that the four descriptive statistics vary over time and have high volatility during the post-COVID-19. More precisely, in March, when the WHO declared COVID-19 a pandemic, the change was more pronounced than in other periods. This phenomenon indicates that the COVID-19 causes an increase of the cryptocurrency’s correlation coefficient or decrease of the cryptocurrency’s information sharing.

### Topological properties of MST and PMFG two methods

Based on the values of *d*_*ρ*_ and the *D*_*I*_ between 102 pairs of cryptocurrencies, we next adopt the MST method issued from Kruskal’s algorithm to build the weighted, connected, and undirected network. We use *Gephi* 0.9.2 [52] to represent these networks. Each cryptocurrency is represented by a labeled node and it is colored by its type classification. Cryptocurrency consists of two main types, token and coin.

Tokens are created on existing blockchains and the most common blockchain token platform is Ethereum, while coins are unique digital currencies that are based on their own, stand-alone blockchains. Each type of cryptocurrency and its subcategories are distinguished in types by using different colors, i.e. mineable coin(orange), non-mineable coin(pink), ethereum(sky blue), and binance chain token(olive). In all networks, we show that the same types of cryptocurrencies have partial connection properties, but are not entirely distinguished by a particular type. This indicates that the type of cryptocurrency does not affect the relevance of cryptocurrency. For the same cryptocurrencies, we also express PMFG based on *d*_*ρ*_ and *D*_*I*_, and we implement these with the following Matlab code written by Aste [53].

**Fig 6:**
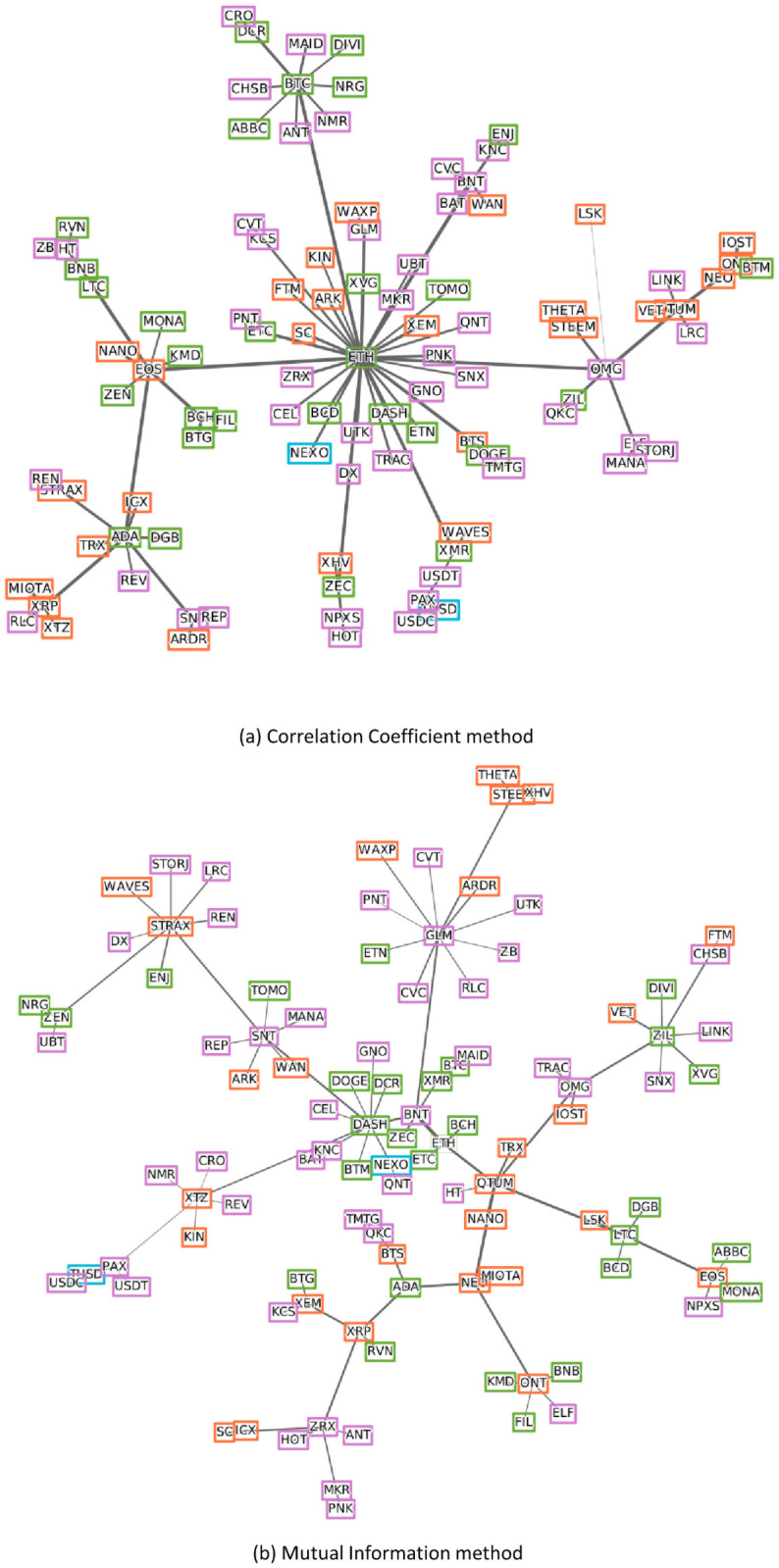
MST for each method in pre-COVID-19 period. Colors represent types (see Table 1) and the edge’s thickness shows inversely proportional to the distance between two nodes.

Fig 6 presents the MST for correlation coefficient and mutual information during the pre-COVID-19 period. According to the correlation coefficient method, it was observed that the most central currency was Ethereum, with more connections than any other and acting in turn as a connector between the other currencies. With Ethereum as a center, 3 cryptocurrency groups are formed around them, such as Bitcoin, EOS, Cardano, while others were linked in a dependent but isolated manner. On the other hand, in the mutual information-based MST, no overwhelmingly dominant node was observed in a tree, so it can be categorized as a distributed network. Golem, Dash, Stratis are slightly more prominent than other currencies. Most of the cryptocurrencies are scattered and expanded.

**Fig 7:**
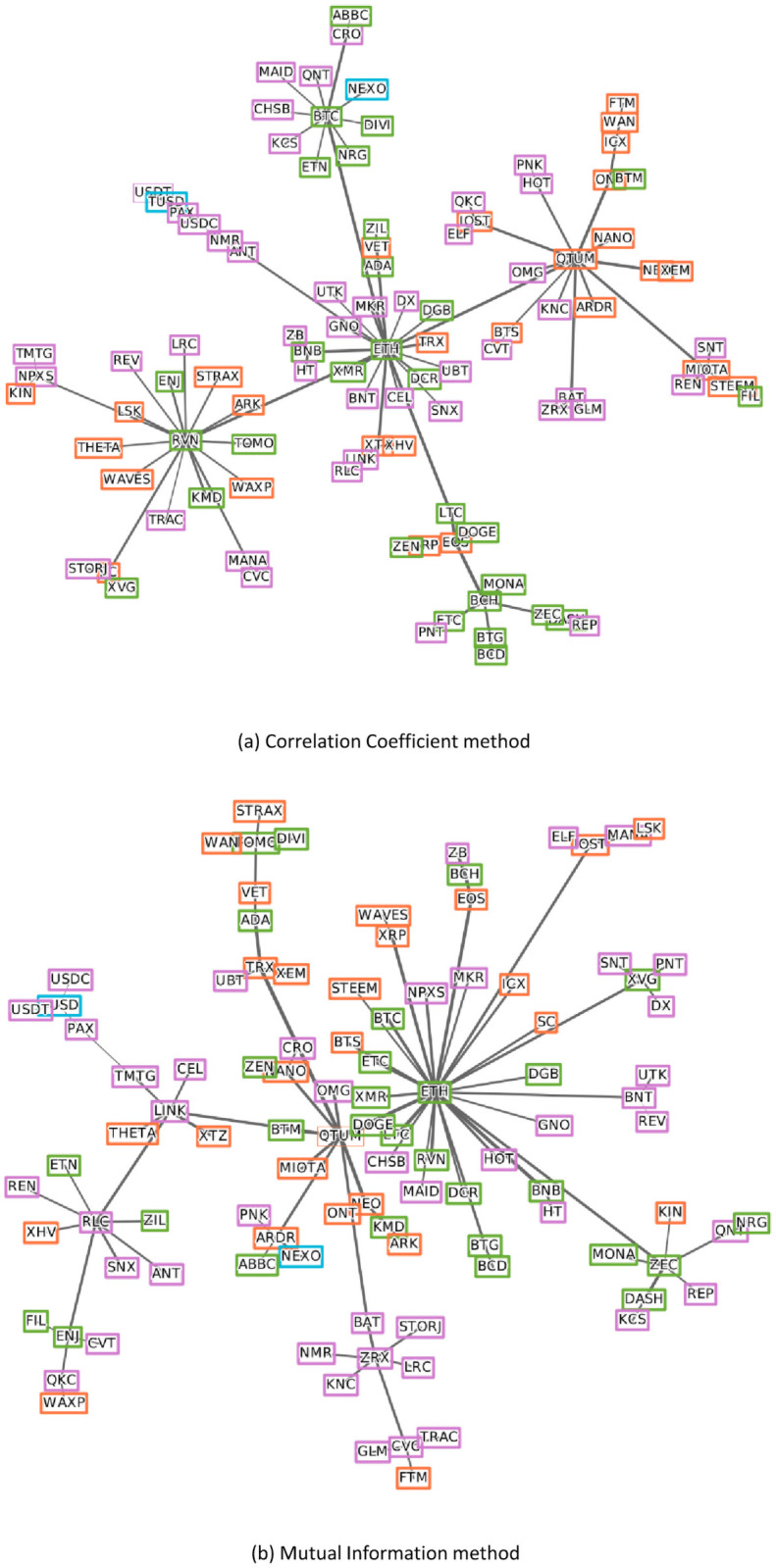
MST for each method in post-COVID-19 period. Colors represent types (see Table 1) and the edge’s thickness shows inversely proportional to the distance between two nodes.

As seen in the network in Fig 7(a), since the COVID-19 outbreak, Ethereum is still dominant but to a lesser extent and Qtum still plays an important role, and the influence of Ravencoin has grown and placed together in the core part of the network. In Fig 7(b), as with the results of the correlation coefficient method, Ethereum takes the leading position, it is now located in the core part of the network with Verge and Qtum and exerting influence over other currencies. It can be seen by comparing the two figures that the results derived using the mutual information method in the post-COVID-19 period are significantly different from the results in the pre-COVID-19 period. We also created PMFGs for comparison of results as shown in S1–S4 Figs. Comparing the four MSTs shows that in the correlation coefficient-based MSTs, most of the cryptocurrencies try to gather around some dominated companies while the other two mutual information-based MSTs are scattered and expanded.

**Fig 8:**
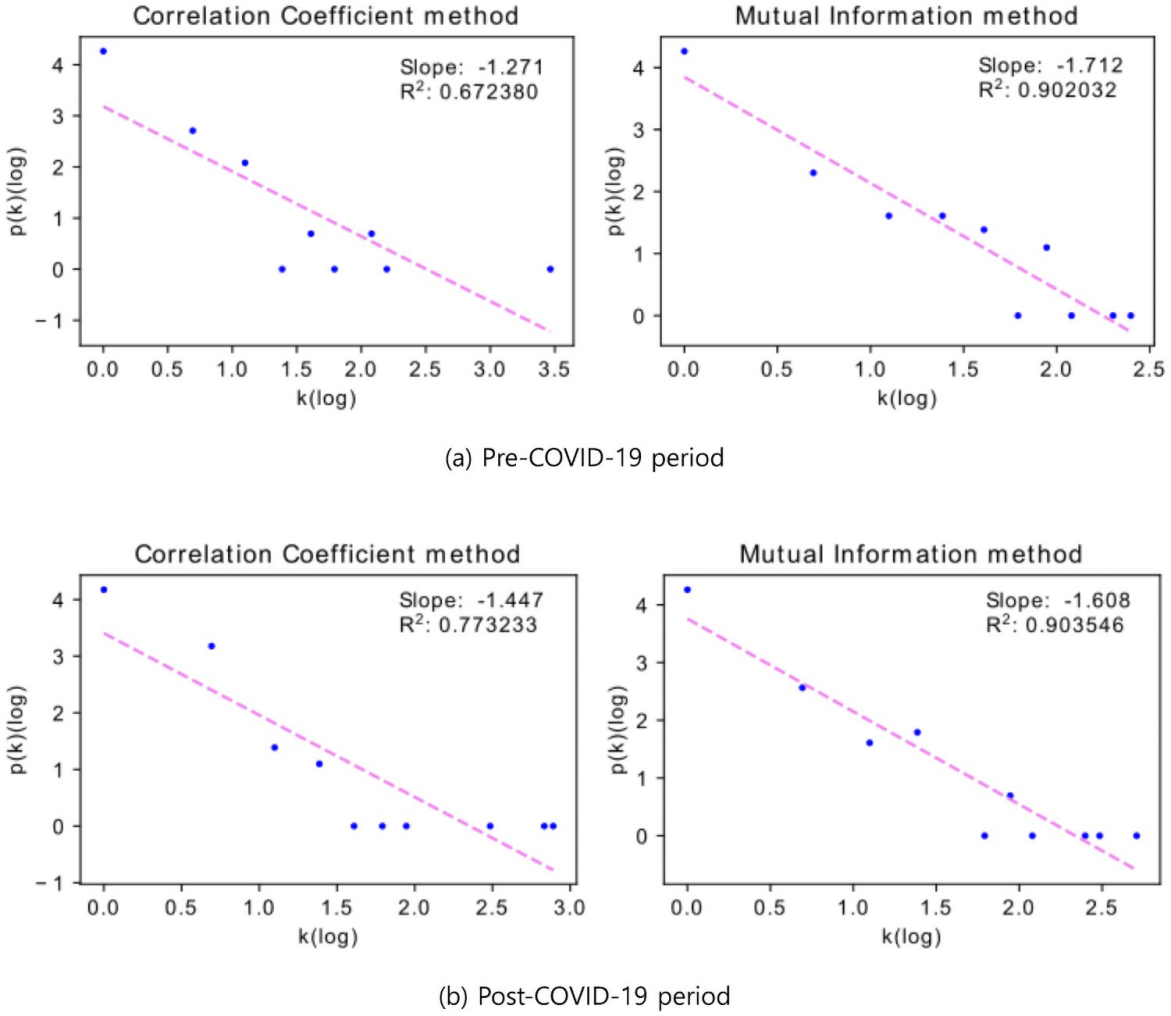
Distribution of nodes’ degrees for the MSTs during pre- and post-COVID periods.

For further exploration, we next investigate the topological properties of the developed networks, including degree distribution, average shortest path length, average clustering coefficient, and diameter. In large-scale real complex network studies, the most important structural characteristics for analyzing patterns are degree distribution. Empirical observations of patterns in the real-world networks have led to the claim that the degree distribution generally follows a single power-law [54, 55]. The degree distribution associated with a network further explains the relationship between establishing edges between nodes, and it is defined as the number of edge connections a single node has. This characteristic helps to define what is known as a power-law graph. The power-law model is a powerful formulation because it directly concludes a statement relating to any node and its degree distribution by representing this relationship exponentially, and it can be expressed as follows: *p*(*k*) ∝ *Ck*^−*α*^ where *k* is node degree, *p*(*k*) is the probability of the *k*-degree nodes, *α* is the power-law exponent, and *C* is a normalization constant. Fig 8 shows that the node degree distribution holds the power-law for MST based on the *d*_*ρ*_ and *D*_*I*_ methods.

First, *α* for the result of applying both methods during the pre-COVID-19 period is 1.271 and 1.712, respectively. And *α* during the post-COVID-19 period is 1.447 and 1.608, respectively. Also, the analysis of Goodness-of-Fit(GOF) of the model is an important aspect. It is commonly measured by R-squared(*R*^2^), a value of *R*^2^ close to 1 indicates the hypothesis of the linear relationship is acceptable. The *R*^2^ for the result of applying both methods during the pre-COVID-19 period is 0.672 and 0.902, respectively. And *R*^2^ during the post-COVID-19 period is 0.773 and 0.904, respectively. These plots show that networks have a scale-free characteristic and through this, it can be seen that the network based on the mutual information method is more effective in representing cryptocurrency behavior than the correlation coefficient method.

To get more insight, we calculated the clustering coefficient for PMFG and also calculated the average shortest path length and diameter for MST. These allow us to see the structural changes in cryptocurrency network in the pre- and post-COVID-19 periods and is presented in Table 2. As can be seen, the mutual information method produces more clustering than the correlation coefficient method, in contrast, the correlation coefficient method requires fewer paths than the mutual information method, and diameter has no tendency by the method.

**Table 2:** Network comparison for PMFGs and MSTs based on correlation coefficient and mutual information.

Period	Network	Average clustering coefficient	Average shortest path length	Diameter
Pre-COVID-19	PMFG *d*_*ρ*_	0.4137	-	-
	MST *d*_*ρ*_	-	4.1197	10
	PMFG *D*_*I*_	0.5278	-	-
	MST *D*_*I*_	-	5.6747	13
Post-COVID-19	PMFG *d*_*ρ*_	0.4314	-	-
	MST *d*_*ρ*_	-	4.3735	14
	PMFG *D*_*I*_	0.5277	-	-
	MST *D*_*I*_	-	5.6414	12

### The results of network’s measures

Since the visual representation of the MST can conceal some common characteristics of the network, using the network measurements defined in the previous section is a good complement to analyzing a network with the shape of its MST.

**Fig 9:**
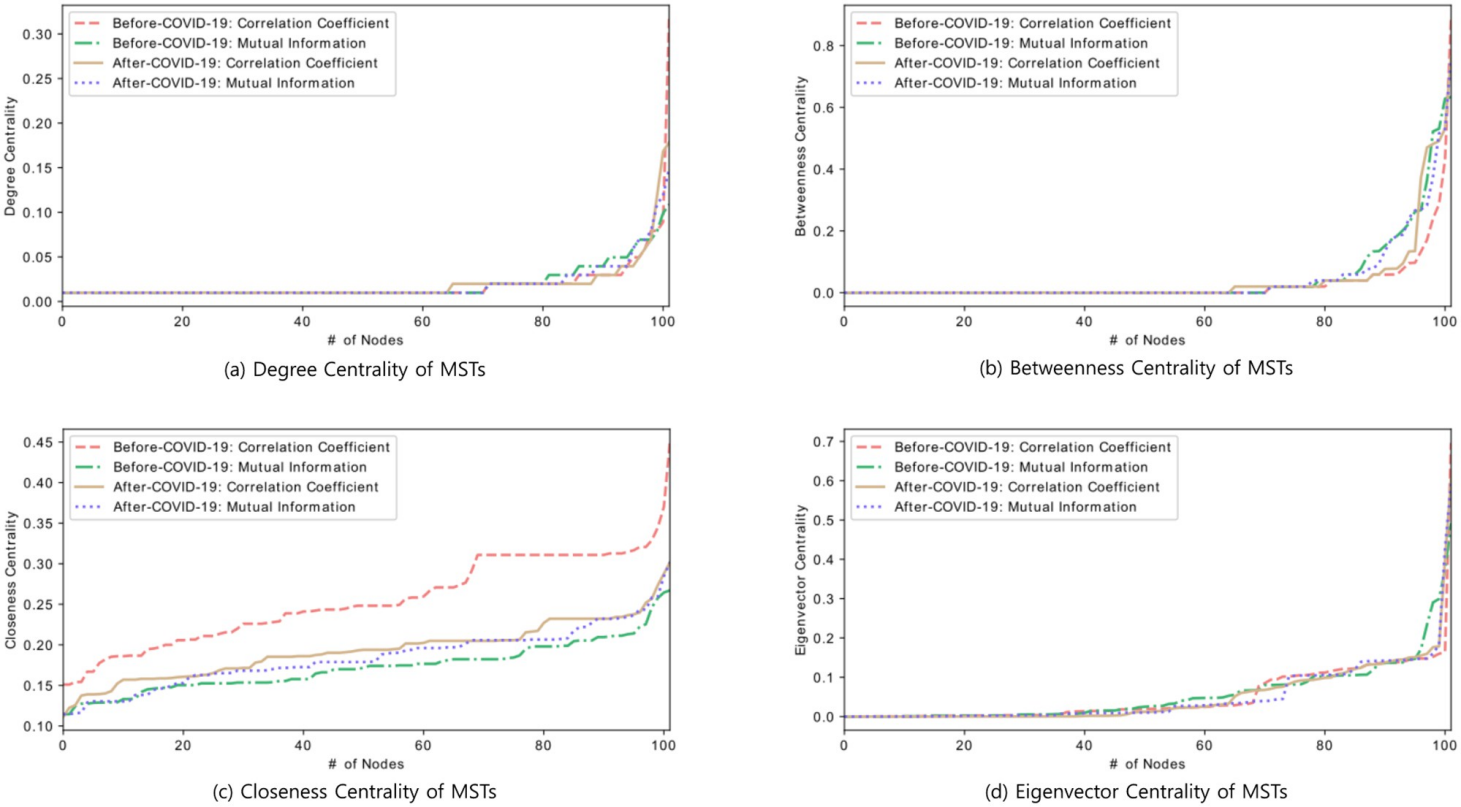
Four types of centrality measures in pre- and post-COVID-19 period.

Fig 9(a) shows the distribution of degree centralities based on correlation coefficient and mutual information method over each period. For both methods of the post-COVID-19 period, the distributions of degree centralities are approximately alike to the distribution of the pre-COVID-19 mutual information method. The pre-COVID-19 correlation coefficient-based method has a particularly high degree of centrality compared to other methods. For the correlation coefficient and mutual information method, the highest degree of centrality during the pre-COVID-19 and post-COVID belongs to Ethereum, Golem, Ethereum, Ethereum respectively. The distribution of degree centrality of the two methods after COVID-19 is almost similar to that of the mutual information method before COVID-19.

A node with higher betweenness centrality would have more effect on the network. As seen in Fig 9(b), the results using the mutual information method show marginally different betweenness centrality for both periods, but the results using the correlation coefficient method show little difference in centrality for the pre- and post-COVID-19 period. Zero betweenness centrality score denotes that no shortest path between any two cryptocurrencies in the network passes through those cryptocurrencies. The result of this method indicates that the number of cryptocurrencies with a betweenness centrality of 0 since the COVID-19 outbreak has decreased from 71 to 65, and the centrality has also increased. The highest score of betweenness belongs to Ethereum, Bancor, Ethereum, and Qtum respectively.

Closeness centrality score in cryptocurrency network exhibits that the more central a currency is, the closer it is to all other currencies. As depicted in Fig 9(c), closeness scores of the nodes during both periods indicate that the results of the mutual information-based method are almost similar, but the results of the correlation coefficient-based method are significantly different. This shows that cryptocurrency shows a higher intimacy in the pre-COVID-19 than period in the post-COVID-19 period. The highest score of closeness belongs to Ethereum, Bancor, Ethereum, and Qtum respectively.

Lastly, eigenvector centrality represents the level of influence of a node within a network. A high eigenvector score of a cryptocurrency demonstrates that it is connected to many nodes with high scores. As shown in Fig 9(d), the distributions of eigenvector centrality are approximately indistinguishable in both periods and in each method. The highest score of closeness belongs to Ethereum, Dash, Ravencoin, and Ethereum respectively.

## Discussion

As it has no theory of cryptocurrency market behavior, there is no frame of reference to clarify whether moving from the well-known method of correlation coefficient to the proposed method of mutual information is rendering the networks closer to reality. In this sense, we can only analyze the resulting networks and find out if they are significantly different, then discover some of their characteristics and add educated guesses based on the properties of the metrics used. The correlation coefficient assumes a linear relationship, and it is easy to understand and easy to model. However, many relationships between two cryptocurrencies are non-linear. In these cases, the correlation coefficient is unnecessarily simplifying the relationship. Moreover, the correlation coefficient does not account for changes over time, it deals with a single number for the entire time period. To overcome these limitations, we apply symbolic time series analysis to continuous data because it is concerned with well-defined measures of uncertainty and complexity. This method uses data symbolization to recognize and express time patterns in complex dynamical processes. In this respect, mutual information based on symbolized data is a more general measure.

The COVID-19 pandemic has seriously affected the global economy and we were able to observe various changes, especially in the stock market [56–58]. Diverse measurements were employed in our work to check any differences between pre- and post-COVID-19 outbreaks, and we concluded that both structure and properties of the networks in these markets have been significantly altered during the pandemic time period. In terms of mutual information-based outcomes, turbulent changes and observed since the COVID-19 outbreak, and the most influential cryptocurrencies have been replaced from Golem, Dash, and Stratis to Ethereum, Verge, and Qtum.

Ethereum is one of the most influential cryptocurrencies in that it can host both other digital tokens or coins and decentralized applications. In particular, in 2020, decentralized finance (DeFi) emerged, and DeFi implanted financial functions into digital ledgers called blockchains, allowing people to do things like lend or borrow funds and earn interest in a savings-like account without the need for traditional intermediaries such as banks. Many DeFi applications are run on the Ethereum blockchain, thus, it can be considered that Ethereum’s influence has grown even more in 2020, after the COVID-19 outbreak.

Verge is a multi-algorithm coin with privacy features, which aims to position itself as an everyday payments tool. The primary reason Verge has grown into an influential coin after the COVID-19 pandemic can be explained by the fact that this cryptocurrency has become favored by major adult sites, and the traffic on that site is increasing significantly during the pandemic.

Lastly, Qtum is a platform that leverages Ethereum smart contract functionality along with the security of the Bitcoin network. Qtum’s Offline Staking was released, this implementation allows user participation, and enables users to stake their Qtum from mobile, hardware, and web wallets. With the new objective of moving a lot of focus to the community, Qtum has also increased its online or social media community engagement, due to COVID-19 and many people staying home because of lockdowns, by hosting several Ask Me Anything sessions, quizzes, and key development updates.

## Conclusion

In this paper, we presented a methodology for creating a cryptocurrency network based on the mutual information method and analyzed the structure of the network before and after the COVID-19 outbreak by constructing an MST and a PMFG for 102 cryptocurrencies along with the correlation coefficient method. The impact of the nonlinear nature of the cryptocurrency market on cryptocurrency prices is considered an important tool in our research and therefore, differs from the most commonly used correlation coefficient methodology in that it processes data based on the method of symbolization of time series. We applied these methods to the market and found that the mutual information method does indeed present a better picture of the market, particularly reconstructing the structure of the market more precisely, while retaining the scale-free property of the networks.

Research on the potential impact of cryptocurrencies is still insufficient when considering the effects of cryptocurrencies on the economy and society. Our future research is to consider a longer timeframe period of integrating the aftermath of the pandemic using more types of cryptocurrencies for optimal analytical purposes. To understand the cryptocurrency market structure more clearly and accurately, we need to further investigate the relationship between price and transaction volume changes in the cryptocurrency market and compare it with other financial markets.

## Supporting information

S1 Fig Planar maximally filtered graphs in pre-COVID-19 using correlation coefficient method. (PNG)

S2 Fig Planar maximally filtered graphs in post-COVID-19 using correlation coefficient method. (PNG)

S3 Fig Planar maximally filtered graphs in pre-COVID-19 using mutual information method. (PNG)

S4 Fig Planar maximally filtered graphs in post-COVID-19 using mutual information method. (PNG)
