# Supplementary figures and images for: The impact of COVID-19 on cryptocurrency markets: A network analysis based on mutual information
Source: PLoS One. 2022 Feb 18;17(2):e0259869. doi: 10.1371/journal.pone.0259869 (PMC8856560; doi:10.1371/journal.pone.0259869)

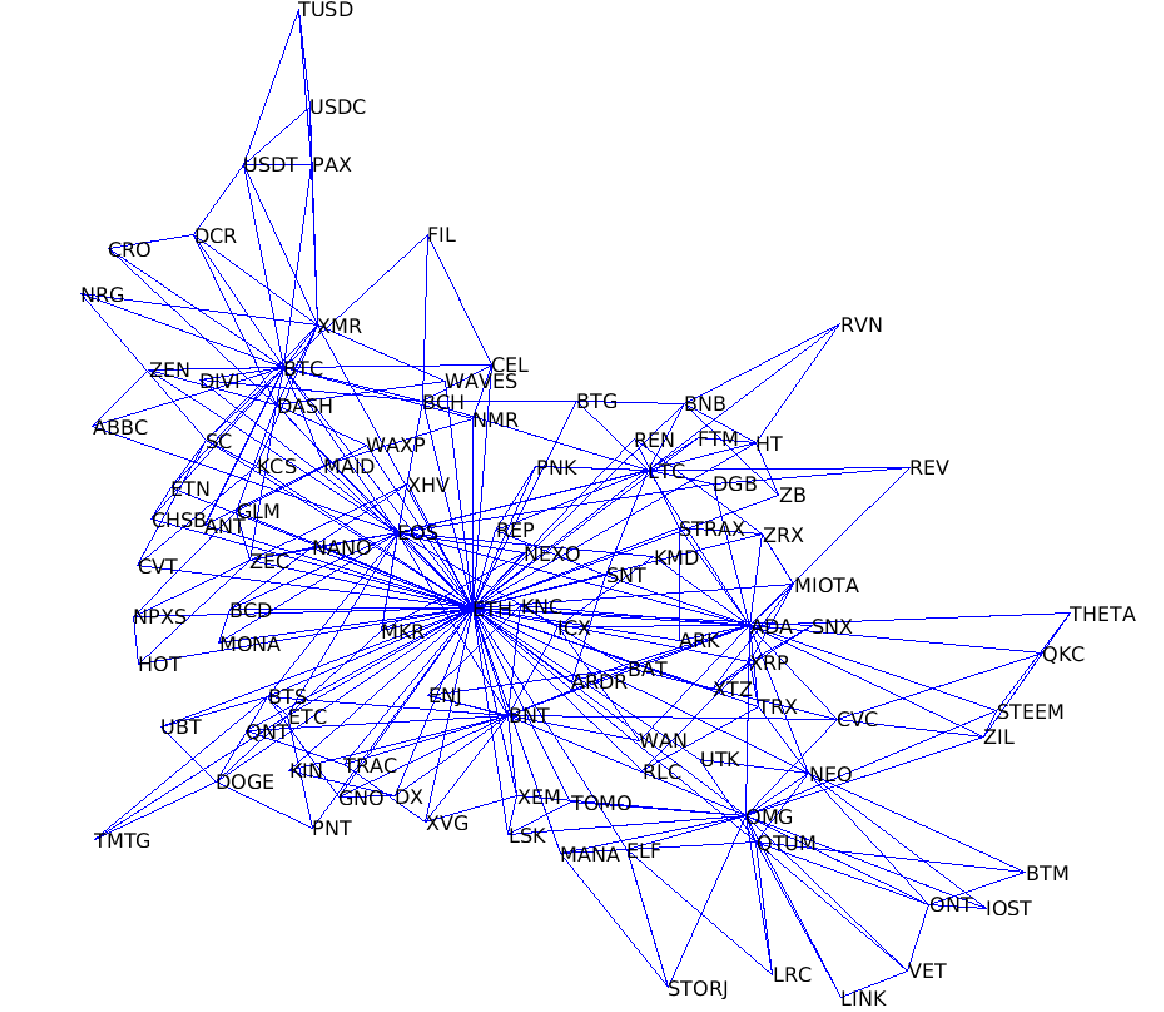

Supplement: S1 Fig — (PNG) [file pone.0259869.s001.png]

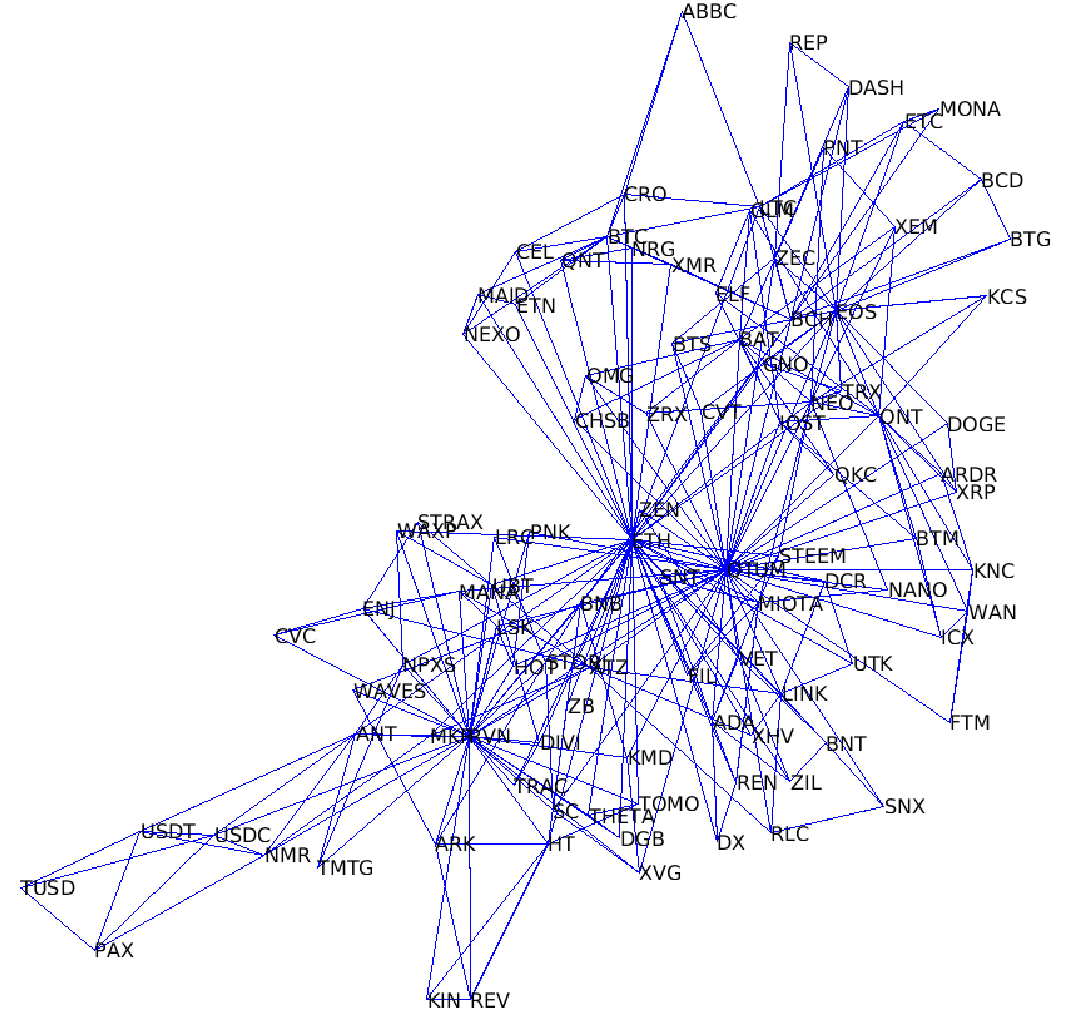

Supplement: S2 Fig — (PNG) [file pone.0259869.s002.png]

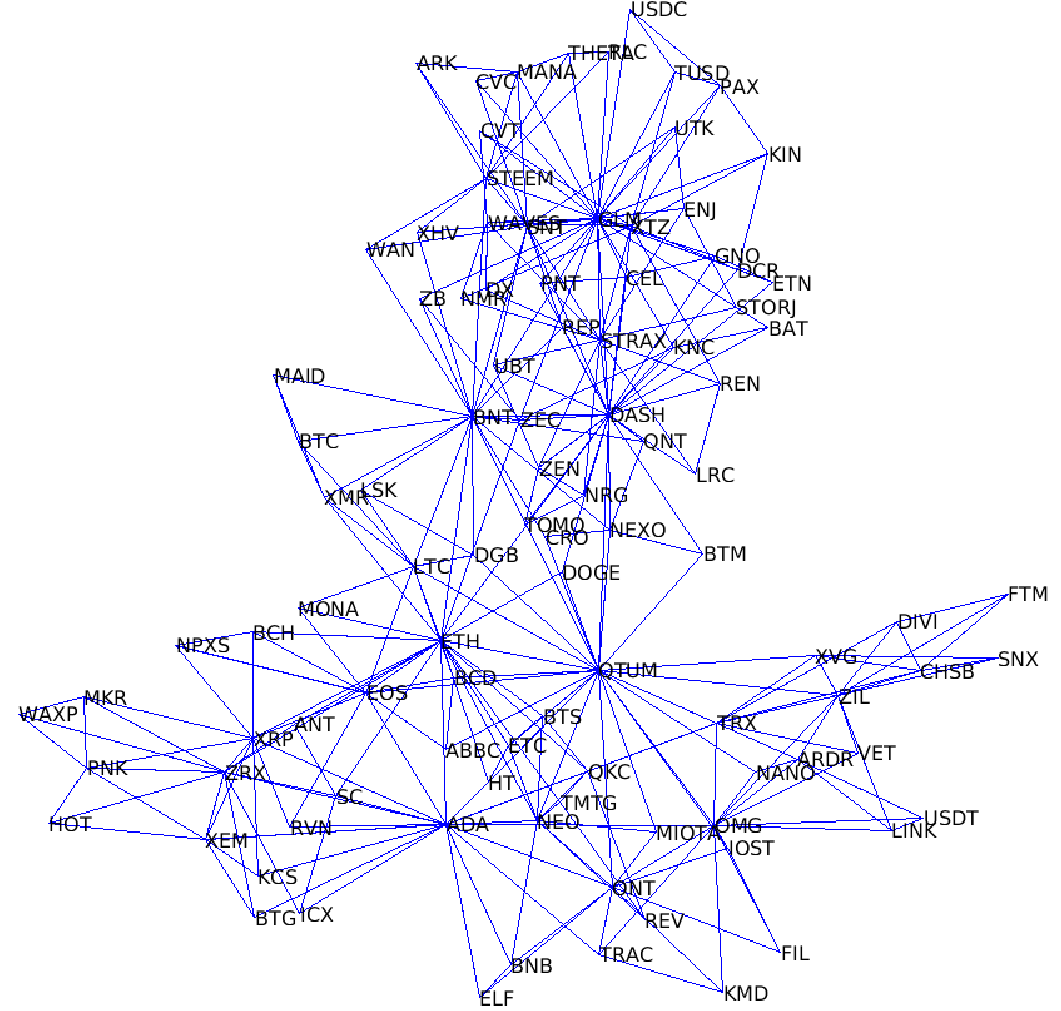

Supplement: S3 Fig — (PNG) [file pone.0259869.s003.png]

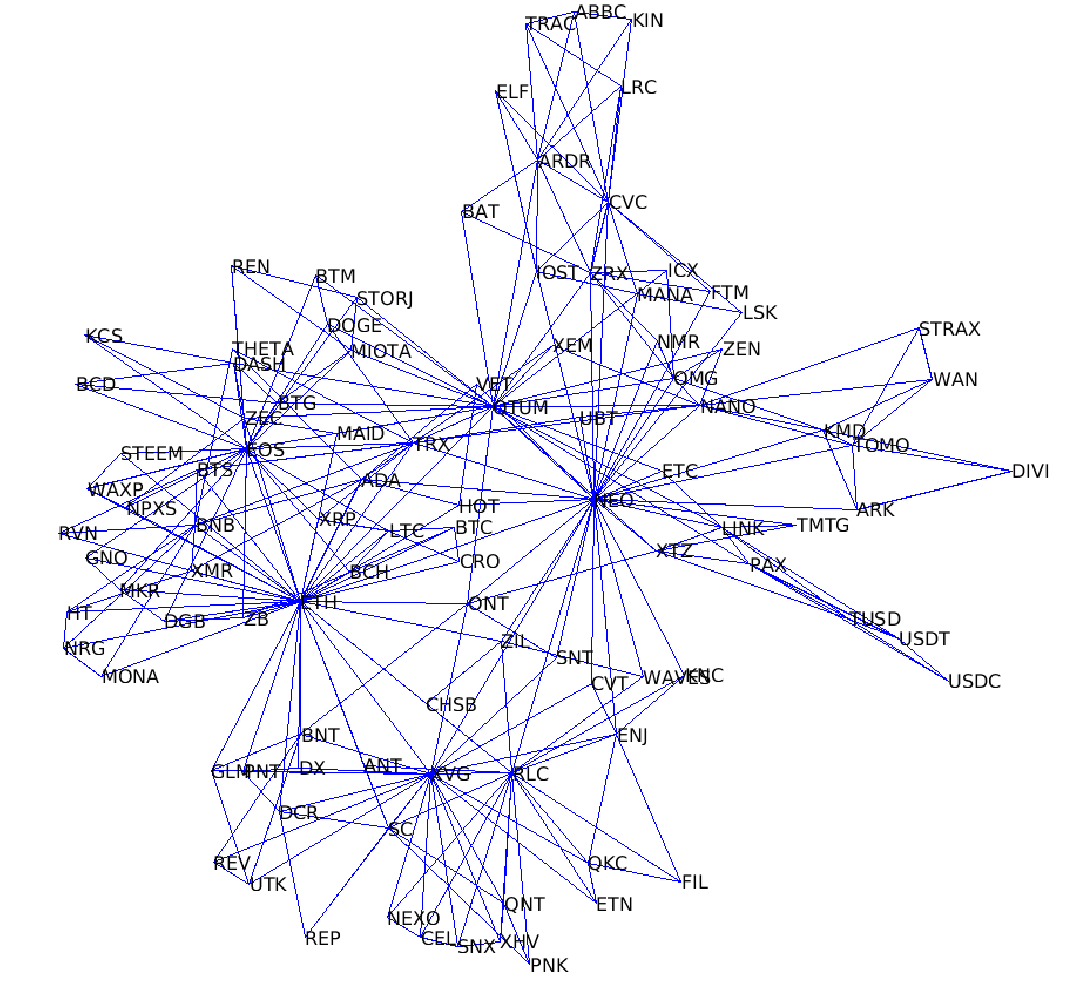

Supplement: S4 Fig — (PNG) [file pone.0259869.s004.png]
